# ZOOMICS: Comparative Metabolomics of Red Blood Cells From Old World Monkeys and Humans

**DOI:** 10.3389/fphys.2020.593841

**Published:** 2020-10-23

**Authors:** Lorenzo Bertolone, Hye K. Shin, Davide Stefanoni, Jin Hyen Baek, Yamei Gao, Evan J. Morrison, Travis Nemkov, Tiffany Thomas, Richard O. Francis, Eldad A. Hod, James C. Zimring, Tatsuro Yoshida, Matthew Karafin, Joseph Schwartz, Krystalyn E. Hudson, Steven L. Spitalnik, Paul W. Buehler, Angelo D’Alessandro

**Affiliations:** ^1^Department of Biochemistry and Molecular Genetics, University of Colorado Denver – Anschutz Medical Campus, Aurora, CO, United States; ^2^Center for Biologics Evaluation and Research, Food and Drug Administration, Silver Spring, MD, United States; ^3^Department of Pathology and Cell Biology, Columbia University, New York, NY, United States; ^4^Department of Pathology, University of Virginia, Charloteseville, VA, United States; ^5^Hemanext, Inc., Lexington, MA, United States; ^6^Blood Center of Wisconsin, Milwaukee, WI, United States; ^7^Department of Pathology and Laboratory Medicine, Milwaukee, WI, United States; ^8^Department of Pathology, University of Maryland School of Medicine, Baltimore, MD, United States; ^9^Department of Pediatrics, Center for Blood Oxygen Transport and Hemostasis, University of Maryland School of Medicine, Baltimore, MD, United States; ^10^Division of Hematology, Department of Medicine, University of Colorado Denver – Anschutz Medical Campus, Aurora, CO, United States

**Keywords:** comparative biology, red blood cell, metabolomics, blood storage, transfusion

## Abstract

As part of the ZOOMICS project, we set out to investigate common and diverging metabolic traits in the blood metabolome across various species by taking advantage of recent developments in high-throughput metabolomics. Here we provide the first comparative metabolomics analysis of fresh and stored human (*n* = 21, 10 males, 11 females), olive baboon (*n* = 20), and rhesus macaque (*n* = 20) red blood cells at baseline and upon 42 days of storage under blood bank conditions. The results indicated similarities and differences across species, which ultimately resulted in a differential propensity to undergo morphological alterations and lyse as a function of the duration of refrigerated storage. Focusing on purine oxidation, carboxylic acid, fatty acid, and arginine metabolism further highlighted species-specific metabolic wiring. For example, through a combination of steady state measurements and ^13^C_6_^15^N_4_-arginine tracing experiments, we report an increase in arginine catabolism into ornithine in humans, suggestive of species-specific arginase 1 activity and nitric oxide synthesis—an observation that may impact the translatability of cardiovascular disease studies carried out in non-human primates (NHPs). Finally, we correlated metabolic measurements to storage-induced morphological alterations via scanning electron microscopy and hemolysis, which were significantly lower in human red cells compared to both NHPs.

## Introduction

Mere scientific curiosity has historically led science to the discovery of novel natural phenomena that turned out to be mechanistically relevant to our understanding of human biology. From Linnaeus’ taxonomical efforts to order nature into separate categories, to modern day DNA-based phylogenetic classifications, scientists have leveraged comparative biology to unravel Nature’s mysteries and learn more about humankind as a species. Embracing this philosophy, we embarked on the ZOOMICS project: by taking advantage of recent developments in high-throughput metabolomics (Nemkov et al., 2017; Reisz et al., 2019), we set out to investigate common and diverging metabolic traits in the blood metabolome across various species. The choice to focus on this matrix and, specifically, on red blood cells (RBCs), stems from an appreciation that blood offers a window into systems metabolism. From classical clinical biochemistry to early clinical metabolomics approaches (D’Alessandro et al., 2012), studies on RBC metabolism have furthered our understanding of systemic responses to aging, inflammation, and physiological (e.g., high altitude) or pathological (e.g., hemorrhage, ischemia) hypoxia. RBC metabolism modulates hemoglobin oxygen binding and off-loading and, in so doing, modulates metabolic activity of the rest of bodily cells that, unlike RBCs, contain mitochondria (Nemkov et al., 2018a). As such, it is unlikely that understanding RBC metabolic variance across multiple species will turn out to be nothing more than a sophisticated exercise in technical metabolomics proficiency. Even if ignoring the relevance of such studies to the standpoint of veterinary medicine, we propose that a metabolomics effort in cataloging RBC metabolomes across species will be relevant to the design and interpretation of research studies that rely on these animals as models for human health and disease.

Animal models have contributed significantly to the fields of hematology and transfusion medicine. Rodent [e.g., mice (Howie et al., 2019), rats (Williams et al., 2019), guinea pigs (Baek et al., 2017)], canine (Klein, 2017), and swine (Clendenen et al., 2017) models have been extensively adopted in blood research for years. However, being genotypically closest to humans, non-human primate (NHP) biology is most phenotypically comparable to human biology. Among all NHPs, baboons (e.g., *Papio anubis*, olive baboon) and macaques (e.g., *Macaca mulatta*, Rhesus macaque, hereon referred to as macaques) are the most frequently studied for biomedical research (VandeBerg et al., 2009; Cox et al., 2013). While both species are equally distant phylogenetically from *Homo sapiens* (Siepel, 2009) (∼93% of DNA sequence homology) (Rhesus Macaque Genome Sequencing and Analysis Consortium, Gibbs et al., 2007; Cox et al., 2013), baboon size and anatomy makes them more phenotypically similar to humans and, thus, preferred for some comparative research studies, including blood research (Valeri and Ragno, 2006). For example, similar to humans, the hematocrit is 39–45% in macaques and 33–46% in baboons (Valeri et al., 1981b), with corresponding Hb levels of 13.1 ± 0.9 and 12.5 ± 0.2 g/dl in male and female macaques, and 12.6 + 1.2 and 12.5 + 1.0 g/dl in male and female baboons (Harewood et al., 1999), respectively (Chen et al., 2009). In addition, RBC distribution widths are 13.0 ± 0.7 and 12.9 + 1.0% in macaques and baboons (Mahaney et al., 2005), respectively, similar to human RBCs (Chen et al., 2009). The mean circulatory life span of macaque RBCs is 98 ± 21 days (Fonseca et al., 2016, 2018), comparable to humans (100–120 days), and significantly longer than mice (55–60 days) (Kaestner and Minetti, 2017). Fresh baboon RBCs also have a similar lifespan to humans (∼100 days) (Valeri et al., 2002), and hydrogen peroxide-damaged baboon RBCs have increased levels of spectrin–hemoglobin complexes facilitating more rapid removal from circulation (McKenney et al., 1990). Further, RBCs in both humans and baboons respond to altitude (D’Alessandro et al., 2016) or hemorrhagic hypoxia (Reisz et al., 2017) by promoting synthesis of 2,3-diphosphoglycerate, thereby enhancing hemoglobin transition state responsiveness to pH and carbon dioxide; this process promotes hemoglobin’s T state conformation (right shift of the hemoglobin oxygen-dissociation curve), oxygen off-loading, and restoration of tissue oxygen homeostasis (Herman et al., 1971).

Studying baboon RBCs in the context of transfusion medicine is not novel. For example, when stored for 3 weeks in citrate phosphate dextrose (CPD) followed by washing, baboon and human RBCs had comparable post-transfusion recoveries (i.e., ∼77%) (Valeri et al., 1981a). This suggested that baboon RBCs were a candidate animal model for blood storage, *in vivo* recovery, and, potentially, for transfusion outcomes. Subsequent studies in 2005 suggested that storing baboon RBCs for 42 days in CPD combined with Additive Solution-1 (i.e., CPD-ADSOL) yielded inferior post-transfusion recoveries, compared to human RBCs. The interspecies differences were even more significant after 49 days of storage, but not at earlier time points (i.e., 74% for both species at storage day 35) (Valeri and Ragno, 2005), suggesting some limitations regarding the translational relevance of baboons as a model for human RBC storage. Further, both young and older baboons demonstrated comparable RBC storage and post-transfusion recoveries based on ^59^Fe-labeling studies (Valeri et al., 1985); interestingly, this observation is not consistent with human data (Tuo et al., 2014) suggesting that RBCs from older donors are more susceptible to the “storage lesion” when compared to those from younger donors. Although not directly related to RBC storage, recent studies have explored the differentiation of baboon-induced pluripotent stem cells (iPSCs) into enucleated mature RBCs (Olivier et al., 2019), as a novel therapeutic approach that may become relevant for transfusion medicine.

Despite several studies, little is known about the metabolism of baboon RBCs, especially how they compare to fresh and stored macaque and human RBCs. Recently, we extensively described the metabolic phenotypes of fresh RBCs from humans and macaques (*Macaca mulatta*), as well as along a weekly continuum in 42-day storage studies (Stefanoni et al., 2020). These experiments identified significant metabolic differences between macaque and human RBCs regarding purine deamination, glutathione metabolism (especially the gamma-glutamyl cycle), arginine metabolism, and membrane phosphatidylserines as a function of storage duration (Stefanoni et al., 2020). Herein, we provide a comparative metabolomics analysis of fresh and stored RBCs from humans and two old-world monkeys, olive baboons (*Papio anubis*) and macaques.

Based on defining RBC metabolic processes that may affect blood storage, disease progression, and translational research, the current work identifies parallel and divergent metabolomics in three primate species (*n* = 20 per group, equally distributed by sex), with several unique and storage-dependent similarities and differences in non- and human–primate RBC metabolism. Notably, a cross-species dimorphism in arginine metabolism was identified in a non-targeted analysis and further validated using stable isotope-labeled arginine tracing. We highlight this pathway, given the importance of nitric oxide (NO) as an RBC-transported signaling molecule that modulates vascular responsiveness to hypoxia (Doctor and Stamler, 2011); in addition, NO depletion may be a relevant component of the storage lesion that affects transfusion efficacy (Bennett-Guerrero et al., 2007; Kanias et al., 2013; Reynolds et al., 2018). Further, in the context of cardiovascular disease (CVD), RBC imbalances in arginase and NO synthase suggest that increased RBC arginase-1 activity decreases NO bioavailability, superoxide production, endothelial dysfunction, and enhances post-ischemic cardiac failure (Yang et al., 2018; Mahdi et al., 2019; Pernow et al., 2019). Nonetheless, to our knowledge no studies have examined RBC metabolomes of multiple, closely related, primate species, particularly regarding arginine metabolism. This “ZOOmics” study of RBC metabolism may critically affect the design and interpretation of CVD studies focusing on NO signaling and metabolism in humans and NHP models (Havel et al., 2017). Such models may be important for studying communication between RBCs and the vasculature in the progression of CVD, and of RBC transfusion quality-based outcomes that are affected by dysregulated metabolic RBC communication.

## Materials and Methods

Since all the methods used in this study have been described in prior work, extensive analytical details and related references to methodological papers and their application to recent RBC storage studies are provided in the Supplementary File—Materials and Methods extended.

### Ethical Statement

All experimental protocols were approved by named institutional committees. Specifically, animal studies were performed according to FDA White Oak Animal Care and Use protocol 2018-31. Human blood was collected under informed consent according to NIH study IRB #99-CC-0168 “Collection and Distribution of Blood Components from Healthy Donors for In Vitro Research Use” under an NIH-FDA material transfer agreement and in compliance with the Declaration of Helsinki.

### Blood Collection, Processing, and Storage

Blood was collected into a syringe using a 20-G needle from the femoral vein of 5-year-old rhesus macaques (*Macaca mulatta—n* = 20; 10 males/10 females) and olive baboons (*Papio anubis*—*n* = 20; 10 males/10 females) under ketamine/dexmedotomidine (7 mg/kg/0.2 mg/kg) anesthesia according to FDA White Oak Animal Care and Use protocol 2018-31. All blood donor macaques originated from the same colony located at Morgan Island, South Carolina, while blood donor olive baboons originated from Southwest National Primate Research Center, San Antonio, Texas, prior to arrival at FDA’s White Oak Campus, Silver Spring, Maryland. Donor blood collections for both species were obtained from basal animals prior to their allocation in other, unrelated studies. Human donor blood was collected into a syringe using a 16-G needle from the median cubital vein of 30- to 75-year-old human volunteers (*n* = 21; 11 males/10 females) under informed consent according to NIH study IRB #99-CC-0168 “Collection and Distribution of Blood Components from Healthy Donors for In Vitro Research Use” under an NIH-FDA material transfer agreement. Blood was collected into acid citrate dextrose, leukofiltered, and stored in AS-3 in pediatric-sized bags designed to hold 20-ml volumes and mimicking the composition of standard full-sized units [i.e., incorporating polyvinylchloride (PVC) and phthalate plasticizers]. RBCs were stored at 4–6°C for 42 days. RBCs and supernatants were separated via centrifugation upon sterile sampling of each unit on days 0, 7, 14, 21, 28, 35, and 42.

### Tracing Experiments With ^13^C_6_^15^N_4_-Arginine

All available RBC lysates from the three species were incubated for 5 min and 24 h at 37°C in AS-3 supplemented with 5 mM stable isotope-labeled ^13^C_6_^15^N_4_-arginine (product no: CNLM-539-H-0.05, Cambridge Isotopes).

### Ultrahigh-Pressure Liquid Chromatography-Mass Spectrometry Metabolomics, Lipidomics, and Arginine Tracing Experiments

A volume of 50 μl of frozen RBC aliquots was extracted 1:10 in ice cold extraction solution (methanol:acetonitrile:water 5:3:2 *v/v/v*) (Reisz et al., 2019). Samples were vortexed and insoluble material pelleted, as described (Nemkov et al., 2016). Analyses were performed using a Vanquish UHPLC coupled online to a Q Exactive mass spectrometer (Thermo Fisher, Bremen, Germany). Samples were analyzed using a 3-min isocratic condition (Nemkov et al., 2017) or a 5-, 9-, and 17-min gradient, as described (Fu et al., 2016; D’Alessandro et al., 2017b). While this method is not directly comparable to classic methods for quantitation of high-energy phosphate compounds (e.g., ATP and DPG), which require acidic extraction, it still affords ac comprehensive overview of (stored) RBC metabolism (Nemkov et al., 2016). For targeted quantitative experiments, extraction solutions were supplemented with stable isotope-labeled standards, and endogenous metabolite concentrations were quantified against the areas calculated for heavy isotopologs for each internal standard (Fu et al., 2016; D’Alessandro et al., 2017b). For arginine tracing experiments, ^13^C and ^15^N tracing into arginine, ornithine, and citrulline was performed as previously described (Seim et al., 2019), through the auxilium of the software El-MAVEN (Agrawal et al., 2019). Graphs and statistical analyses (either *t*-test or repeated measures ANOVA) were prepared with GraphPad Prism 8.0 (GraphPad Software, Inc, La Jolla, CA, United States), GENE E (Broad Institute, Cambridge, MA, United States), and MetaboAnalyst 4.0 (Chong et al., 2018). Extensive details for this section are provided in the Supplementary File—Materials and Methods extended.

### Hemolysis Measurements

Percent hemolysis was measured based on % hematocrit, supernatant hemoglobin (Hb) (g/dl), and total (Hb) (supernatant + RBC, g/dl) in 50-μl samples obtained weekly from storage bags. Supernatant and RBCs were separated using a hematocrocrit centrifuge (Thermo Fisher, Frederick, MD, United States). Hematocrit was recorded, and supernatant was separated from RBCs. Supernatant and lysed RBC Hb levels were measured using a Carey 60 UV-visible spectrophotometer (Agilent Technologies, Santa Clara, CA, United States). Oxy ferrous Hb (HbFe^2+^O_2_) and ferric Hb (HbFe^3+^) concentrations were determined based on the extinction coefficients for each species. Molar extinction coefficients used to calculate Hb concentrations in heme equivalents were: 15.2 mM^–1^ cm^–1^ at 576 nm for Hb(O_2_) and 4.4 mM^–1^ cm^–1^ at 631 nm for ferric Hb using 50 mM potassium phosphate buffer, pH 7.0 at ambient temperature, in both cases. Total heme was calculated by adding these values and converting (heme) (microM) to total (Hb) (g/dl).

### Red Blood Cell Morphological Evaluation

Red blood cells were fixed (1% glutaraldehyde in 0.1M phosphate buffer) and post-fixed with 1% osmium tetroxide for 1 h at room temperature, prior to further preparation and evaluation by scanning electron microscopy, as described (Baek et al., 2018).

## Results

### The Metabolic Phenotypes of Fresh RBCs From NHPs Are More Similar to Each Other Than to Humans

**FIGURE 1:**
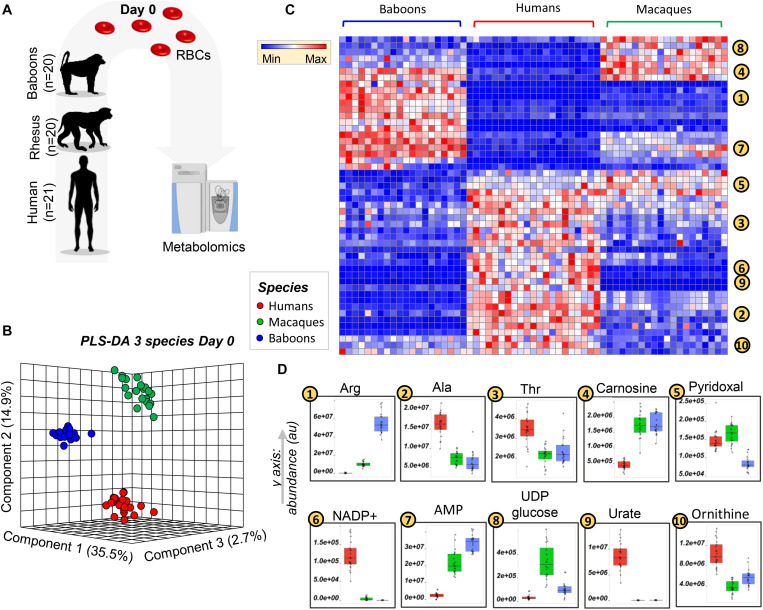
Metabolic phenotypes of freshly drawn human (red), macaque (green), and baboon (blue) red blood cells (RBCs) were tested via ultrahigh-pressure liquid chromatography-mass spectrometry (UHPLC-MS)-based metabolomics approaches **(A)**. Partially supervised partial least square-discriminant analysis (PLS-DA; **B**) and hierarchical clustering analysis **(C)** of the metabolomics data indicate a significant difference between human and NHP RBCs (PC1: 35.5% of the total variance), and between macaque and baboon RBCs (PC2: 14.9% of the total variance; **B**). **(D)** Several representative metabolites, among the top significant changes at baseline, across species, as determined by ANOVA.

Metabolomics analyses were performed on freshly drawn human (*n* = 21), baboon (*n* = 20), and macaque (*n* = 20) RBCs (Figure 1A). All the raw data are extensively reported in tabulated form as Supplementary Table 1 and as a heat map in Supplementary Figure 1. Partial least square-discriminant analysis (PLS-DA; Figure 1B—Supplementary Table 1 also includes a comparison between PCA and PLS-DA, details of PLS-DA performances, and validation) and hierarchical clustering analyses (Figure 1C) were performed on these data, showing that RBC metabolomes of the NHPs were separated from those of humans across principal component (PC) 1, which explained 35.5% of the total variance (Figure 1B). This observation is clearer in the heat map in Figure 1C, which shows the top 50 significant metabolites by ANOVA from cross-species comparisons. Some metabolites (alanine, threonine, ornithine, NADP^+^, and urate) were consistently higher in human RBCs, whereas AMP and carnosine were significantly lower in humans compared to NHPs (Figure 1D). We also observed metabolic divergences between baboons and macaques. For example, pyridoxal was significantly lower in baboons, whereas macaques had comparable levels to humans (Figure 1D). In other cases, metabolic differences that we had previously reported in the comparison of macaques and humans (Stefanoni et al., 2020) (e.g., arginine), were further increased in baboon RBCs and significantly greater than macaques (Figure 1D). Other metabolites (e.g., UDP-glucose in the hexosamine pathway) were higher in macaque RBCs than in humans or baboons, which had similar levels of this metabolite (Figure 1D).

### Species-Specific Metabolic Differences Were Enhanced by Storage Under Blood Bank Conditions

**FIGURE 2:**
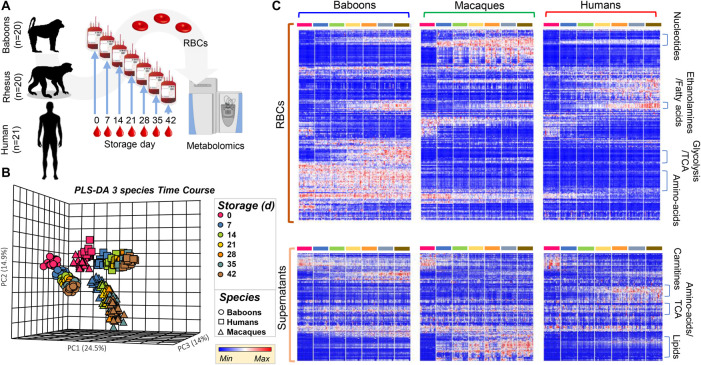
Metabolic phenotypes of human (red), macaque (green), and baboon (blue) RBCs and supernatants during refrigerated storage under blood bank conditions **(A)**. Units were tested weekly, from storage day 0 until the end of the shelf-life (storage day 42). Partially supervised PLS-DA **(B)** and hierarchical clustering analysis **(C)** of the metabolomics data indicate a significant difference between human and non-human primate (NHP) RBCs (PC1: 24.5% of the total variance), and between macaque and baboon RBCs (PC2: 14.9%) and storage duration (14%; **B**). In **(C)**, heat maps indicate significant metabolic changes during storage across species, as determined by repeated measures ANOVA, for RBCs (top) and supernatants (bottom).

Red blood cell storage in blood banks is critically important for therapeutic purposes; however, RBC storage induces many metabolic changes affecting energy and redox metabolism, membrane function, tissue oxygenation, and cellular communication (Yoshida et al., 2019). Because these pathways differed in humans, compared to NHPs, in fresh RBCs, we hypothesized that they would be further aggravated by storage for 42 days, the shelf-life of packed RBCs in the United States and most European countries. Metabolomics analyses were performed on samples collected weekly by sterile docking of units from Storage Day 0 through 42 (Figure 2A). Analyses were performed on 854 samples [*n* = 61 (20 baboons, 20 macaques, and 21 humans) each for seven storage time points for RBCs and supernatants], which were clustered on the basis of their metabolic phenotypes in the PCA (Figure 2B). PC1 showed a significant impact of storage duration (from left to right—storage days 0–42; Figure 2B), explaining 24.5% of the total variance. However, PC2 and PC3 still explained > 14% of the residual variance, with human RBCs clustering closer to macaques than baboons throughout storage.

In the heat map in Figure 2C, the most significant metabolites and time-dependent %hemolysis comparisons were evaluated by repeated measures two-way ANOVA (Supplementary Table 1) and were grouped by general metabolic pathways. Further comparisons between baboon and human %hemolysis were evaluated using a Student’s *t*-test or a by one-way ANOVA with Holm–Sidak’s multiple-comparison test to compare %hemolysis across the three species. A vectorial version of this figure is provided as Supplementary Figures 2, 3 for RBCs and supernatants, along with metabolite names. Major patterns in these time-series data as a function of the species were identified via ANOVA simultaneous component analysis (ASCA—Supplementary Table 1). Some of the metabolites that are differentially impacted across species as a function of storage are representatively grouped into classes in Figure 2C. While intracellular fatty acids and phosphatidylethanolamines were comparable across species during storage, supernatant levels of lipids were the lowest in humans and highest (and increased during storage) in macaques; nonetheless, baboons unexpectedly had the highest levels of carnitine-conjugated fatty acids in the supernatants (Figure 2C).

**FIGURE 3:**
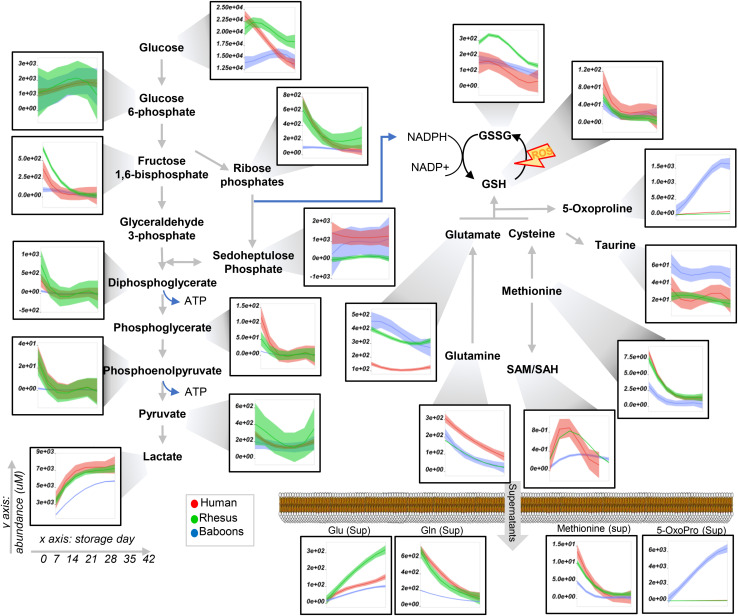
Glycolysis and pentose phosphate pathway, glutathione, and methionine metabolism in human (red), macaques (green), or baboon (blue) RBCs from storage days 0 to 42. Line plots indicate median (*n* = 20) + quartile ranges for all the groups. Y axis represents peak areas (arbitrary units) normalized to the median of week 0 measurement for each independent species. Extensive statistical analyses, including fold changes and repeated measures ANOVA, are provided in Supplementary Table 1.

### Species-Specific Metabolic Changes Throughout Storage

**FIGURE 4:**
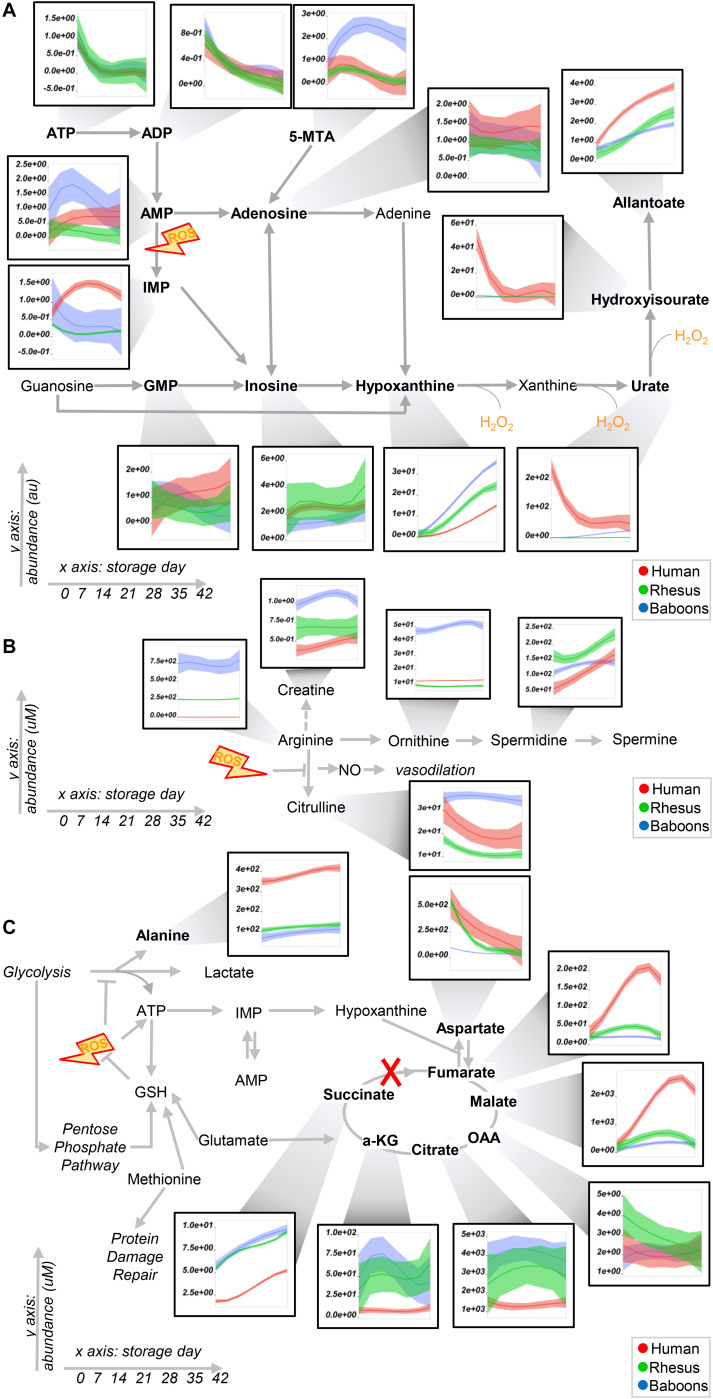
Purine **(A)** and arginine **(B)** metabolism, transaminases, and carboxylic acids **(C)** in human (red), macaque (green), or baboon (blue) RBCs from storage days 0 to 42. Line plots indicate median (*n* = 20) + quartile ranges for all the groups. Y axis represents peak areas (arbitrary units) normalized to the median of week 0 measurement for each independent species. Extensive statistical analyses, including fold changes and repeated measures ANOVA are provided in Supplementary Table 1.

**FIGURE 5:**
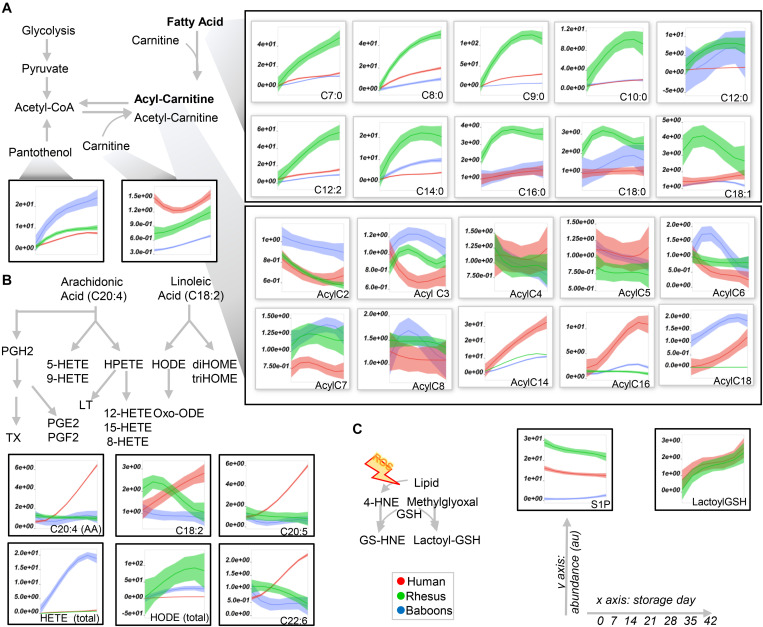
Fatty acid (top), acyl-carnitine (bottom; **A**), eicosanoids **(B)**, and glyoxylate and sphingosine 1-phosphate (S1P) metabolism in human (red), macaque (green), or baboon (blue) RBs from storage days 0 to 42. In **(A**,**B)**, abbreviations indicate the length in carbon atoms (**C**) of the fatty acid chain and the number of desaturations. The prefix “Acyl-” is used to denote acyl-carnitines in **(A)**. Line plots indicate median (*n* = 20) + quartile ranges for all the groups. Y axis represent peak areas (arbitrary units) normalized to the median of week 0 measurement for each independent species. Extensive statistical analyses, including fold changes and repeated measures ANOVA are provided in Supplementary Table 1.

To expand on the unsupervised analyses described above, line plots (median + quartile ranges) were plotted for human (red), baboon (blue), and macaque (green) RBCs for different pathways, including glycolysis and glutamine/glutathione metabolism (Figure 3), purine, arginine, and carboxylic acid/transaminase metabolism (Figures 4A–C, respectively), and fatty acids and acyl-carnitines (Figure 5). Detailed statistics including fold changes, ANOVA, and ASCA analyses are reported in Supplementary Table 1.

Baboon RBCs had the lowest levels of glucose, glycolytic, and pentose phosphate pathway intermediates and byproducts (lactate and ribose phosphate isomers) throughout storage, whereas humans had the highest (though not significantly higher than macaques; Figure 3). Human RBCs had the highest levels of reduced glutathione (GSH) and the lowest of oxidized glutathione (GSSG), which was significantly higher in macaques compared to baboons (Figure 3). In contrast, baboons had the highest levels of 5-oxoproline, an activation marker of the gamma-glutamyl cycle (Figure 3). Compared to humans, baboons and macaques both had significantly higher markers of glutaminolysis (low extracellular glutamine, high intracellular or extracellular glutamate for baboons and macaques, respectively). Further alterations in sulfur metabolism were noted in NHPs. For example, levels of the antioxidant taurine were highest in baboons throughout storage; however, methionine and *S*-adenosylmethionine levels were significantly lower in baboons, suggesting a decreased capacity to repair isoaspartyl damage compared to human and macaque RBCs (Figure 3). Analysis of purine metabolism revealed significantly higher levels of methylthioadenosine in baboons, compared to the other species, suggesting an increased activation of salvage pathway *S*-adenosyl metabolites. Conversely, humans had the highest levels of deaminated purine products: IMP, urate [a potent antioxidant (Tzounakas et al., 2015), which was barely detectable in NHPs], 5-hydroxyisourate, and allantoate, with the notable exception of hypoxanthine, which was highest in NHPs (Figure 4A). Although we previously reported an effect of sex on this pathway in macaques and humans (Stefanoni et al., 2020) (e.g., the hypoxanthine guanosine phosphoribosyl transferase gene X-linked), only minor, but significant, sex-associated differences were noted in hypoxanthine and allantoate levels in baboons (female > male) during storage (Supplementary Figure 4A).

The most notable metabolic change was that baboons had the highest levels of arginine, citrulline, ornithine, and creatine (Figure 4B), with ornithine/arginine (a marker of arginase activity) (D’Alessandro et al., 2019a) and citrulline/arginine ratios (a marker of nitric oxide synthase activity) (Kleinbongard et al., 2006) being higher in females and males, respectively (Supplementary Figure 4B). Despite having the lowest levels of arginine, human RBCs consistently had the second highest levels of citrulline throughout storage and the highest citrulline/arginine ratios (a marker of nitric oxide synthase activity). To validate this observation further, fresh RBC lysates from these species were incubated with 5 mM ^13^C_6_^15^N_4_-arginine for 5 min and 24 h at 37°C (Supplementary Figure 5A). As expected, no residual urea cycle activity was observed in any species (i.e., no isotopolog ^13^C_5_^15^N_2_-citrulline was detected), consistent with the absence of mitochondria in mature RBCs. However, significant arginase activity was seen in humans (^13^C_5_^15^N_2_-ornithine detected as > 80% of the total after 24 h in humans—Supplementary Figure 5C) and nitric oxide synthase activity in all species—in particular, macaques (Supplementary Figure 5C), were observed, resulting in the generation of ^13^C_6_^15^N_3_-citrulline. Overall, when normalized to the total levels of labeled arginine detected as a percentage of the total (Supplementary Figure 5C), our results indicate that human RBCs have significantly higher arginase activity, and lower nitric oxide synthase activity, than macaques and baboons.

Human RBCs also had the highest transaminase activity (as inferred by the levels of the metabolic products of the activity of these enzymes—e.g., alanine, aspartate—and consumption of glutamate), but the lowest levels of carboxylic acid products of transamination reactions (e.g., alpha-ketoglutarate and related citrate and succinate), which were comparable between NHPs (Figure 4C). On the other hand, the highest levels of fumarate and malate in human RBCs are consistent with increased purine deamination and, perhaps, salvage in comparison to NHPs (Figure 4C). Finally, human RBCs had the highest levels of free carnitine and long-chain acyl-carnitines (14 carbon atoms or longer) throughout storage, followed by baboons (Figure 5A), especially males (Supplementary Figure 6). Although macaque RBCs had significantly lower levels of acyl-carnitines throughout storage, they had the highest levels of free fatty acids, from 7 to 18 carbon atoms (Figure 5A). In contrast, human RBCs had increased levels of polyunsaturated fatty acids and lower levels of oxylipins, which were highest in baboons [hydroxyeicosatetraenoic acids (HETEs)] and macaques [hydroxyoctadecenoic acids (HODEs)] (Figure 5B). Macaque and human RBCs had the highest levels of sphingosine 1-phosphate (Figure 5C).

### Human RBCs Are Characterized by Lower Storage Hemolysis and Morphological Alterations

**FIGURE 6:**
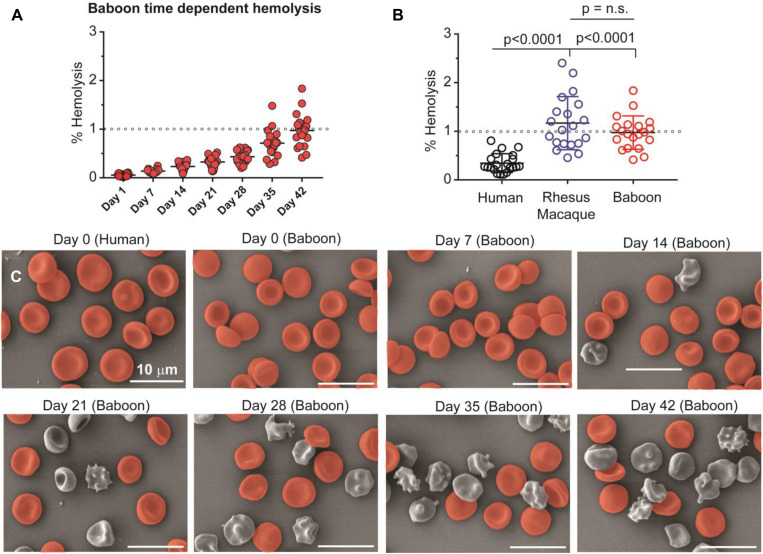
Spontaneous hemolysis as a function of storage duration in baboons **(A)** and an interspecies comparison of end of storage hemolysis **(B)**. NHPs have significantly higher end-of-storage hemolysis than humans, with individual macaques showing the most variability and the greatest maximum individual hemolysis levels. Collectively, mean hemolysis levels between baboons and macaques were similar (*p* = 0.231, n.s.). Scanning electron micrographs **(C)** are shown for human (day 0) and baboon (weekly) RBCs as a function of storage duration (discocytes in red; echinocytes, spheroechinocytes, acanthocytes, stomatocytes, and spherocytes in gray). Days 0 and 42 equivalent micrographs for human and macaque RBCs are provided as Supplementary Figure 6.

Considering the differences in lipid metabolism in stored RBCs and supernatants across these species, we anticipated a lower rate of vesiculation and a lower severity of the storage lesion (based on RBC morphology and hemolysis *in vitro*) with human RBCscompared to NHPs. Storage hemolysis increased progressively in baboons, with more than half demonstrating hemolysis levels ≥ 1% at storage day 42 (i.e., above the Food and Drug Administration quality threshold; Figure 6A). This was similar in baboons and macaques, whereas all human RBC samples had 42-day hemolysis values of < 1% (Figure 6B). By scanning electron microscopy, by storage day 42, > 70% of baboon RBCs had lost their discocytic phenotype (Figure 6C). Macaque RBCs were similar, with 80% loss of the discocytic phenotype by storage day 42 (Supplementary Figure 7). Unlike the NHPs, human RBCs had a better-preserved morphology, with > 70% discocytes on storage day 42 (Supplementary Figure 7), consistent with the patterns of storage hemolysis across these three species.

### Common and Divergent Metabolic Correlates to Species-Specific Hemolysis

**FIGURE 7:**
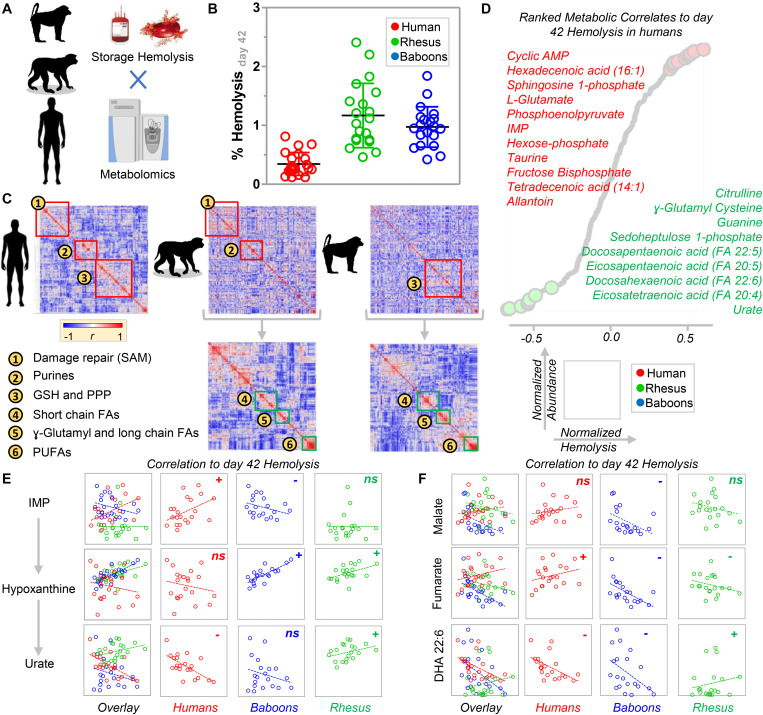
Determination of species-specific metabolic correlates to end of storage hemolysis in baboons, macaques, and humans **(A)**. **(B)** A side-by-side comparison of end of storage hemolysis across the three species. Significantly lower hemolysis was observed in human RBCs. **(C)** Metabolic linkage analysis (i.e., correlation of metabolites to metabolites) in humans, macaques, and baboons [the latter two maintaining the clustered order from human metabolic linkage analyses (i.e., top) or performing species-specific correlations (i.e., bottom)]. These analyses highlighted groups of metabolites and related metabolic pathways that are preserved between humans and macaques, humans and baboons, or baboons and humans. **(D)** A ranked list of metabolic correlates to hemolysis in humans. Based on the analyses described above, a few representative metabolites were plotted that significantly correlated to hemolysis in one species, but not (or inversely correlated) in both of the other two species tested, including purine oxidation metabolites **(E)**, carboxylic acids, and polyunsaturated fatty acids **(F)**.

We performed correlation analyses (Figure 7A) of end of storage metabolites with storage hemolysis (Figure 7B). This metabolic linkage analysis (D’Alessandro et al., 2017a) provides further, unsupervised, data-driven insights into the metabolic wiring of RBCs evaluated in this study and provides additional correlative evidence of their potential contributions to RBC hemolysis mechanisms. Thus, in human and macaque RBCs, strong correlations were preserved between the metabolite levels involved in *S*-adenosylmethionine-dependent protein damage repair mechanisms, purine metabolism (except for urate), and glutathione and pentose phosphate pathway homeostasis (Figure 7C). In contrast, human RBCs were more comparable to baboons regarding short-chain fatty acid metabolism, whereas long-chain and polyunsaturated fatty acid metabolism was similar between macaques and baboons (Figure 7C, bottom half). These observations are consistent with our prior findings identifying several polyunsaturated fatty acids among the top negative correlates of storage hemolysis (Figure 7D). Therefore, this was further leveraged in Figure 7C to rank species-specific metabolic correlates to hemolysis (in tabular form in Supplementary Table 1). Notably, as in our prior studies, deaminated purines, including IMP, positively correlated with hemolysis in human RBCs, while urate negatively correlated (Figure 7E). However, this observation was only partially recapitulated in baboons and macaques, with a positive correlation of hypoxanthine with hemolysis in both species, but a negative correlation with the hypoxanthine precursor, IMP, or the downstream oxidation product, urate, in baboon and macaque RBCs, respectively (Figure 7E). This suggests species-specific biochemistry (at the structural, expression, or functional level) in xanthine dehydrogenase oxidase, AMP deaminase 3 (AMDP3), or hypoxanthine guanosine-phosphoribosyltransferase (HGPRT). Although no species-specific polymorphisms were noted around the active sites in AMPD3 (99% homology across species) and HGPRT (100% sequence homology), some potentially relevant polymorphisms around the active site (residues 803E and 881R) were seen in XDH, despite ∼97% sequence homology across the three species (Supplementary Figure 8). Similar changes in correlation trends to storage hemolysis across these species were seen with carboxylic acids, such as malate and fumarate (positive correlation with hemolysis in humans, but negative correlations in NHPs). Additionally, polyunsaturated fatty acids negatively correlated with hemolysis in human and baboon RBCs, but were positively correlated in macaques (Figure 7F).

## Discussion

The present study describes significant metabolic, morphological, and storage hemolysis changes between fresh and stored RBCs from human donors and old-world monkeys, specifically olive baboons and rhesus macaques. Baseline metabolic changes were further enhanced by refrigerated storage under standard blood bank conditions. In prior studies, we showed that RBCs undergo alterations to energy and redox metabolism during refrigerated storage (Cancelas et al., 2015; Rolfsson et al., 2017; D’Alessandro et al., 2018). The so-called metabolic “storage lesion” varies across blood donors (D’Alessandro et al., 2019b), with RBCs from some donors being more susceptible to hemolysis *in vitro* or upon oxidant, mechanical, or osmotic challenge applied at the end of their shelf-life (Kanias et al., 2017). The metabolic storage lesion ultimately impacts the tissue metabolome of healthy autologous transfusion recipients (D’Alessandro et al., 2019a) or in sickle-cell disease patients undergoing RBC exchange transfusion (Gehrke et al., 2019). These observations were phenocopied in rodent models of blood storage and transfusion (Howie et al., 2019), providing evidence *in vivo* of the dysregulation of RBC viability, recovery, systemic iron exposure, and redox metabolism in the initial 24 h after transfusion (Gehrke et al., 2019). Further, storage-induced alteration of purine deamination, and subsequent metabolism by AMP deaminase 3 and xanthine dehydrogenase/oxidase, predicted the ability of transfused RBCs to circulate in the recipient (Nemkov et al., 2018b). Although prestorage levels of urate, a major circulating antioxidant, were associated with improved RBC storage quality (Tzounakas et al., 2015), the levels of its precursor (i.e., hypoxanthine) were negatively associated with the preservation of energy metabolism in stored RBCs and predict accelerated RBC removal post-transfusion (Nemkov et al., 2018b). In the present study, metabolites of the purine deamination pathway were the most divergent when comparing human and NHP RBCs; human RBCs had an order of magnitude higher levels of urate both at baseline and after refrigerator storage. Of note, although such differences had been reported in plasma studies (Misra et al., 2018) involving baboons, the impact of this RBC phenotype change had not been described in relation to RBC storage quality. This observation can be explained by species-specific peculiarities in urate metabolism, which would end up affecting the circulating levels of this metabolite and, as a consequence, RBC urate concentration at the time of blood drawn. In addition, since one of the enzymes involved in hypoxanthine recycling into guanosine (i.e., HGPRT) is encoded by an X-linked gene, it is interesting to note a marginal, but significant, effect of sex on hypoxanthine accumulation across the primate species evaluated here and in previous comparisons between human and macaque RBCs (Stefanoni et al., 2020).

A possible explanation of the divergencies in this pathway across these species is provided by appreciating the polymorphisms in the genes coding for xanthine oxidase in old-world monkeys, compared to humans, as indicated by polymorphisms that mapped within 20 amino acids the active sites of this enzyme (E803 and R881). Increases in carboxylic acid metabolites in macaque and baboon RBCs, compared to humans, along with increases in 5-methylthioadenosine, are consistent with greater purine salvage reactions in NHPs, which may explain the observed decreases in purine deamination products.

Prior studies of macaque RBC storage observed alterations in free fatty acids, acyl-carnitines, and phosphatidylserines, compared in stored human RBCs. Herein, we expanded on these findings by confirming increases in storage-induced hemolysis and morphologic changes in this species, compared to humans. In addition to confirming similar metabolic, hemolytic, and morphological dysregulation in baboon RBCs, we identified unique alterations of lipid oxidation and remodeling (e.g., via carnitine conjugation and the Lands cycle) (Wu et al., 2016) between baboons and macaques. Alterations in this pathway (especially with respect to fatty acyl-compositions) may at least be in part explained by species-specific diets (Stefanoni et al., 2020).

Perhaps one of the most translationally relevant observations in this study is the divergent metabolism of arginine in human RBCs, compared to baboons and macaques. Arginine conversion to citrulline by an RBC-specific nitric oxide synthase (Kleinbongard et al., 2006) was proposed to contribute to system-wide NO metabolism and, thus, vasodilation. Conversely, arginine metabolism by arginase 1, which is also present and functional in mature RBCs (D’Alessandro et al., 2019a), generates ornithine without the net production of vasoactive NO. Increased arginase-1 activity in the RBCs of patients with Type 2 diabetes is associated with superoxide production and accelerated endothelial dysfunction (Yang et al., 2018; Mahdi et al., 2019; Pernow et al., 2019), suggesting a link between increased RBC arginase-1 activity and CVD. We previously showed that macaque RBCs had significantly higher levels of arginine and its catabolites, compared to human RBCs. To understand this observation further, herein we combined steady state measurements and metabolic tracing with ^13^C^15^N-arginine to show that baboon RBCs have an order of magnitude higher levels of arginine, creatine, and ornithine than macaque RBCs. However, human RBCs had significantly higher steady state levels of citrulline than macaques and significantly higher labeled ornithine (suggestive of higher arginase activity), compared to both NHPs. This observation is critical in light of the central role of this pathway in cardiovascular studies, suggesting caution when interpreting (Havel et al., 2017) CVD studies focusing on NO signaling in baboons and macaques. In addition, these observations cannot be explained by polymorphisms in arginase 1 (>98% sequence homology across the three species) or NO synthase. On the other hand, expression levels and/or activity of arginase may vary across these species as a result of regulatory alterations that has evolved under positive pressure, as classic comparative biology studies report a 957 + 206 μmol urea/g of hemoglobin/h arginase activity in human RBCs compared to < 1 in *Papio* baboon species (Spector et al., 1985). Moreover, sex dimorphisms in this pathway that were seen previously in human tissues (Reckelhoff et al., 1998) and RBCs (Contreras-Zentella et al., 2019), and macaque RBCs (Stefanoni et al., 2020), are also observed in baboons.

Finally, levels of amino acids involved in transamination reactions were increased in human RBCs, compared to NHPs, with increased glutaminolysis and glutamate consumption in stored human RBCs as storage progressed. Similarly, human and macaque RBCs showed comparable levels of methionine (a marker of oxidant stress and protein isoaspartyl damage-repair in mature RBCs) (Reisz et al., 2018). In contrast, baboon RBCs coped with oxidant stress through activating the gamma-glutamyl cycle (5-oxoproline levels were two orders of magnitude higher in this species compared to macaques and humans) and taurine, a dietary antioxidant that only lower mammals (e.g., rodents, cats) are thought to be capable of synthesizing (Ripps and Shen, 2012).

## Conclusion

Despite the observational nature of the study, we provide the first comparative metabolomics analysis of fresh and stored human, baboon, and macaque RBCs. The results indicated similarities and differences across species, which ultimately resulted in a differential propensity to undergo morphological alterations and lyse as a function of the duration of refrigerated storage. Focusing on purine oxidation and carboxylic acids, fatty acids, and arginine metabolism, we further highlighted species-specific metabolic wiring (e.g., increased arginine catabolism into citrulline and ornithine in humans) and correlations with storage-induced hemolysis. Notably, while RBCs from these NHPs had been reported to have similar *in vivo* lifespan to human RBCs (Valeri et al., 2002; Kaestner and Minetti, 2017), the increased susceptibility to hemolysis during storage may result from the appreciation that current storage additives have been optimized for human RBCs, a caveat that may be relevant for future transfusion medicine studies using NHPs as a model.

## Data Availability Statement

All datasets generated for this study are included in the article/Supplementary Material.

## Ethics Statement

The studies involving human participants were reviewed and approved by NIH study IRB #99-CC-0168 “Collection and Distribution of Blood Components from Healthy Donors for In Vitro Research Use” under an NIH-FDA material transfer agreement and in compliance with the Declaration of Helsinki. The patients/participants provided their written informed consent to participate in this study. The animal study was reviewed and approved by FDA White Oak Animal Care and Use protocol 2018-31.

## Author Contributions

HS, JB, and PB collected and stored the samples. TY, SS, PB, and AD’A provided the essential materials and methods to perform the study. PB and YG performed the hemolysis and SEM evaluation of RBCs. LB, EM, DS, TN, and AD’A performed the metabolomics analyses (untargeted and targeted quantitative) and tracing experiments. LB and AD’A performed the data analysis and prepared the figures and tables. AD’A, PB, and SS wrote and modified the first draft of the manuscript, which was revised by all the other authors, including MK, JS, TT, JZ, EH, RF, and KH. All the authors contributed to the finalizing of the manuscript.

## Conflict of Interest

Though unrelated to the contents of this manuscript, the authors declare that AD’A and TN are founders of Omix Technologies, Inc., and Altis Biosciences LLC. AD’A and SS are consultants for Hemanext, Inc. SS is also a consultant for Tioma, Inc. PB is a consultant for KaloCyte, Inc. AD’A and JZ are consultants for Rubius Inc. AD’A is a consultant for Forma, Inc. The remaining authors declare that the research was conducted in the absence of any commercial or financial relationships that could be construed as a potential conflict of interest.
