# Supplementary material for: ZOOMICS: Comparative Metabolomics of Red Blood Cells From Old World Monkeys and Humans
Source: Front Physiol. 2020 Oct 23;11:593841. doi: 10.3389/fphys.2020.593841 (PMC7645159; doi:10.3389/fphys.2020.593841)
Supplement: Supplementary file 1 [file Data_Sheet_1.PDF]

Supplementary Figures for

ZOOMICS: Comparative Metabolomics of Red Blood Cells from Old World Monkeys and Humans

Lorenzo Bertolone,[1] Hye Kyung H. Shin,[2] Davide Stefanoni,[1] Jin Hyen Baek,[2] Yamei Gao,[2] Evan J Morrison,[1] Travis Nemkov,[1] Tiffany Thomas,[3] Richard O. Francis,[3]Eldad A. Hod,[3] James C. Zimring,[4] Tatsuro Yoshida,[5] Matthew Karafin,[6] Joseph Schwartz,[3] Krystalin Hudson,[3] Steven L. Spitalnik,[3] Paul W. Buehler,[7,8*,] Angelo D'Alessandro[1,9,*,**]**

1) Department of Biochemistry and Molecular Genetics, University of Colorado Denver – Anschutz Medical Campus, Aurora, CO, USA
2) Center for Biologics Evaluation and Research, Food and Drug Administration, Silver Spring, MD, USA.
3) Department of Pathology & Cell Biology, Columbia University, New York, NY, USA
4) Department of Pathology, University of Virginia, Charloteseville, VA, USA
5) Hemanext Inc, Lexington, MA, USA
6) Blood Center of Wisconsin, Milwaukee, Wisconsin. & Department of Pathology & Laboratory Medicine, Milwaukee, Wisconsin.
7) Department of Pathology, University of Maryland School of Medicine, Baltimore, MD, USA
8) Department of Pediatrics, Center for Blood Oxygen Transport and Hemostasis, University of Maryland School of Medicine, Baltimore, MD, USA
9) Department of Medicine – Division of Hematology, University of Colorado Denver – Anschutz Medical Campus, Aurora, CO, USA

***Corresponding authors:**

Angelo D'Alessandro, PhD
Department of Biochemistry and Molecular Genetics
University of Colorado Anschutz Medical Campus
12801 East 17th Ave., Aurora, CO 80045
Phone # 303-724-0096
E-mail: angelo.dalessandro@ucdenver.edu

Paul W. Buehler, Pharm.D, PhD
Center for Blood Oxygen Transport and Hemostasis
University of Maryland School of Medicine
670 W. Baltimore Street
HSF III, 8th Floor, Room 8180
Baltimore, MD 21201
E-mail: pbuehler@som.umaryland.edu

SUPPLEMENTARY METHODS - EXTENDED

Ethical statement All experimental protocols were approved by named institutional committees. Specifically, animal studies were performed according to FDA White Oak Animal Care and Use protocol 2018-31. Human blood was collected under informed consent according to NIH study IRB #99-CC-0168 "Collection and Distribution of Blood Components from Healthy Donors for In Vitro Research Use" under an NIH-FDA material transfer agreement.

Blood collection, processing and storage: Blood was collected into a syringe using a 20 G needle from the femoral vein of five-year-old rhesus macaques (*Macaca mulatta* - n=20; 10 male/10 female) and olive baboons (*Papio anubis* – n=20; 10 male/10 female) under ketamine/dexmedotomidine (7 mg/kg/0.2 mg/kg) anesthesia according to FDA White Oak Animal Care and Use protocol 2018-31. All blood donor rhesus macaques originated from the same colony located at Morgan Island, South Carolina, while blood donor olive baboons originated from Southwest National Primate Research Center, San Antonio Texas prior to arrival at FDA's White Oak Campus, Silver Spring, Maryland. Donor blood collections for both species were obtained from basal animals prior to their allocation in other, unrelated studies. Human donor blood was collected into a syringe using a 16 G needle from the median cubital vein of 30-75-year-old human volunteers (n=21; 11 male/10 female) under informed consent according to NIH study IRB #99-CC-0168 "Collection and Distribution of Blood Components from Healthy Donors for In Vitro Research Use" under an NIH-FDA material transfer agreement. Blood was collected into acid citrate dextrose, leukofiltered, and stored in AS-3 in pediatric-sized bags designed to hold 20 ml volumes and mimicking the composition of standard full-sized units (i.e., incorporating polyvinylchloride (PVC) and phthalate plasticizers). RBCs were stored at 4-6ºC for 42 days. RBCs and supernatants were separated via centrifugation upon sterile sampling of each unit on days 0, 7, 14, 21, 28, 35, and 42.

Tracing experiments with $^{13}C_6$ $^{15}N_4$-Arginine

All available RBC lysates from the three species were incubated for 5 min and 24h at 37ºC in AS-3 supplemented with 5 mM stable-isotope labeled $^{13}C_6$ $^{15}N_4$-Arginine (product no: CNLM-539-H-0.05, Cambridge Isotopes).

RBC storage and sampling: RBCs were stored at 4-6ºC for 42 days. Sampling was performed in a biosafety cabinet, by obtaining 500 µL from each unit using a 16G/1-inch needle and 1 ml syringe after thorough mixing of each bag. Collections were performed on days 0, 7, 14, 21, 28, 35, and 42. Samples were centrifuged at 2,500 rpm for 10 minutes at 4ºC, separated into supernatant and RBCs for each time point, and stored at -80ºC until analysis.

Sample processing and metabolite extraction: A volume of 50 µL of frozen RBC aliquots was extracted 1:10 in ice cold extraction solution (methanol:acetonitrile:water 5:3:2 *v/v*).[1,2] Samples were vortexed for 30 min at 4ºC and insoluble material pelleted via centrifugation for 10 min at 10,000g at 4ºC, as described.[1,2] For lipidomics analyses,[3–5] supernatants were diluted 1:1 (v/v) with 10 mM ammonium acetate for analysis by ultra-high pressure liquid chromatography coupled to mass spectrometry (UHPLC-MS).

Ultra-High-Pressure Liquid Chromatography-Mass Spectrometry (MS) metabolomics: The analytical platform employs a Vanquish UHPLC system (Thermo Fisher Scientific, San Jose, CA, USA) coupled online to a Q Exactive mass spectrometer (Thermo Fisher Scientific, San Jose, CA, USA).

Metabolomics: UHPLC-MS metabolomics analyses were performed as described,[3,5,6] using a Vanquish UHPLC system coupled online to a high-resolution Q Exactive mass spectrometer (Thermo Fisher, Bremen, Germany). Samples were resolved over a Kinetex C18 column (2.1x150 mm, 1.7 µm; Phenomenex, Torrance, CA, USA) at 45°C. A volume of 10 ul of sample extracts was injected into the UHPLC-MS. Each sample was injected and run four times with two different chromatographic and MS conditions as follows: 1) using a 5 minute gradient at 450 µL/minute from 5-95% B (A: water/0.1% formic acid; B:acetonitrile/0.1% formic acid) and the MS was operated in positive mode and 2) using a 5 minute gradient at 450 µL/minute from 5-95% B (A: 5% acetonitrile, 95%water/1 mM ammonium acetate; B:95%acetonitrile/5% water, 1 mM ammonium acetate) and the MS was operated in negative ion mode. The UHPLC system was coupled online with a Q Exactive (Thermo, San Jose, CA, USA) scanning in Full MS mode at 70,000 resolution in the 60-900 m/z range, 4 kV spray voltage, 15 sheath gas and 5 auxiliary gas, operated in negative or positive ion mode (separate runs). These chromatographic and MS conditions were applied for both relative and targeted quantitative metabolomics measurements, with the differences that for the latter targeted quantitative post hoc analyses were performed on

the basis of the stable isotope-labeled internal standards used as a reference quantitative measurement, as detailed below.

Lipidomic:s Samples were resolved as described,[4–6] over an ACQUITY HSS T3 column (2.1 x 150 mm, 1.8 µm particle size (Waters, MA, USA) using an aqueous phase (A) of 25% acetonitrile and 5 mM ammonium acetate and a mobile phase (B) of 50% isopropanol, 45% acetonitrile and 5 mM ammonium acetate. Samples were eluted from the column using either the solvent gradient: 0-1 min 25% B and 0.3 mL/min; 1-2 min 25-50% B and 0.3 mL/min, 2-8 min 50-90% B and 0.3 mL/min, 8-10 min 90-99% B and 0.3 mL/min, 10-14 min hold at 99% B and 0.3 mL/min, 14-14.1 min 99-25% B 1and 0.3 mL/min, 14.1-16.9 min hold at 25% B and 0.4 mL/min, 16.9-17 min hold at 25% B and resume flow of 0.3 mL/min. isocratic elution of 5% B flowed at 250 µl/min and 25ºC or a gradient from 0- 5% B over 0.5 min; 5-95% B over 0.6 min, hold at 95% B for 1.65 min; 95-5% B over 0.25 min; hold at 5% B for 2 min, flowed at 450 µl/min and 35ºC[7]. The Q Exactive mass spectrometer (Thermo Fisher Scientific, San Jose, CA, USA) was operated independently in positive or negative ion mode, scanning in Full MS mode (2 µscans) from 150 to 1500 m/z at 70,000 resolution, with 4 kV spray voltage, 45 shealth gas, 15 auxiliary gas.

MS2 analyses for untargeted metabolomics For discovery mode untargeted metabolomics, dd-MS2 was performed at 17,500 resolution, AGC target = 1e5, maximum IT = 50 ms, and stepped NCE of 25, 35 for positive mode, and 20, 24, and 28 for negative mode, as described in Gehrke et al. J Protome Research 2019,[8] and applied to similar samples (i.e., stored RBCs) in Stefanoni et al. Haematologica 2019[5] and D'Alessandro et al. Haematologica 2020[6].

Quality control and data processing: Calibration was performed prior to analysis using the Pierce™ Positive and Negative Ion Calibration Solutions (Thermo Fisher Scientific). Acquired data was then converted from .raw to .mzXML file format using Mass Matrix (Cleveland, OH, USA). Samples were analyzed in randomized order with a technical mixture (generated by mixing 5 ul of all samples tested in this study) injected every 15 runs to qualify instrument performance. This technical mixture was also injected three times per polarity mode and analyzed with the parameters above, except CID fragmentation was included for unknown compound identification (10 ppm error for both positive and negative ion mode searches for intact mass, 50 ppm error tolerance for fragments in MS2 analyses – further details about the database searched below).

Metabolite assignment and relative quantitation: Metabolite assignments, isotopologue distributions, and correction for expected natural abundances of deuterium, ^{13}C, and ^{15}N isotopes were performed using MAVEN (Princeton, NJ, USA),[9] against an in house library of deuterated lipid standards (SPLASH® LIPIDOMIX® Mass Spec Standard, Avanti Lipids) and in house libraries of 3,000 unlabeled (MSMLS, IROATech, Bolton, MA, USA; IroaTech ; product A2574 by ApexBio; standard compounds for central carbon and nitrogen pathways from SIGMA Aldrich, St Louis, MO, USA) and labeled standards (see below for the latter). Discovery mode analysis was performed with standard workflows using Compound Discoverer 2.1 SP1 (Thermo Fisher Scientific, San Jose, CA). From these analyses, metabolite IDs or unique chemical formulae were determined from high-resolution accurate intact mass, isotopic patterns, identification of eventual adducts (e.g., Na+ or K+, etc.) and MS^2 fragmentation spectra against the KEGG pathway, HMDB, ChEBI, and ChEMBL databases. Additional untargeted lipidomics analyses were performed with the software LipidSearch (Thermo Fisher, Bremen, Germany).

Statistical Analysis: Graphs and statistical analyses (either t-test or repeated measures ANOVA) were prepared with GraphPad Prism 5.0 (GraphPad Software, Inc, La Jolla, CA), GENE E (Broad Institute, Cambridge, MA, USA), and MetaboAnalyst 4.0.[10] In MetaboAnalyst, relative quant data (but not for abs quant), raw values for integrated peak areas for each metabolite were normalized on a pool of day 0 controls and auto-scaled for each species independently prior to margining all the data for multivariate analysis, including principal component analysis, partial least square discriminant analysis and repeated measures ANOVA – as in prior studies on RBC storage in humans[6] and other species[5].

Targeted quantitative metabolomics: Targeted quantitative metabolomics analyses were performed on all RBC and supernatant samples from fresh and stored human, baboon and RM RBCs. All the samples were extracted with the exact same protocol detailed above. However, for this validation analysis, extraction solutions (methanol:acetonitrile:water 5:3:2 *v/v*) were supplemented with the standards detailed in the table below:

Metabolite	Labeling	Neutral Formula	MW	Vendor	Product ID	Final concentration (uM)
a-Ketoglutarate	13C5	*C5H6O5	151.05	Cambridge	CLM-2411-PK	1
Adenosine						1
Citric acid	2,2,4,4-D4	C6H4D4O7	196.15	Cambridge	DLM-3487-PK	1
Fumaric acid	1,4-13C2	*C2C2H4O4	118.05	Cambridge	CLM-4454-PK	1
Glutathione (GSH)	Gly13C2,15N	*C2C8H17*N1N2O[illegible]	310.3	Cambridge	CNLM-6245.50	10
Glucose	13C6	*C6H12O6	186.09	Cambridge	CLM-1396-1	10
Lactate	1-13C	*C1C2H6O3	113.05	Cambridge	CLM-1577-1	40
Palmitic acid	U-13C16	*C16H32O2	272.25	Cambridge	CLM-409-0.5	1
Sphingosine 1P	D7	C18H31D7NO5P	386.51	Avanti	860659-1MG	0.5
Succinic acid	13C x 4	*C4H6O4	122.05	Cambridge	CLM-1571-0.25	1
AA Mix (including the standards below)				Cambridge	MSK-A2-1.2	1
L-Alanine	13C3, 15N	*C3H7*NO2				1
L-Arginine:HCl	13C6, 15N4	*C6H15Cl*N4O2 (isotopic *C6H14*N4O2)				1
L-Aspartic acid	13C4, 15N	*C4H7*NO4				1
L-Cystine	13C6, 15N2	*C6H12*N2O4S2				1
L-Glutamic acid	13C5, 15N	*C5H9*NO4				1
Glycine	13C2, 15N	*C2H5*NO2				1
L-Histidine:HCl:H2O	13C6, 15N3	*C6H12Cl*N3O3 (isotopic *C6H9*N3O2)				1
L-Isoleucine	13C6, 15N	*C6H13*NO2				1
L-Leucine	13C6, 15N	*C6H13*NO2				1
L-Lysine:2HCl	13C6, 15N2	*C6H16Cl2*N2O2 (isotopic *C6H14*N2O2)				1
L-Methionine	13C5, 15N	*C5H11*NO2S				1
L-Phenylalanine	13C9, 15N	*C9H11*NO2				1
L-Proline	13C5, 15N	*C5H9*NO2				1
L-Serine	13C3, 15N	*C3H7*NO3				1
L-Threonine	13C4, 15N	*C4H9*NO3				1
L-Tyrosine	13C9, 15N	*C9H11*NO3				1
L-Valine	13C5, 15N	*C5H11*NO2				1

Samples were processed with the same UHPLC-MS setup described above. However, upon acquisition, raw data were converted to .mzxml and peak areas for light and heavy (stable isotope-labeled) isotopologues were exported for each metabolite, as extensively described in prior methodological work[1,3,11]. In details, absolute concentrations were determined using the following formula: [light] = (abundance light) / (abundance heavy) * [heavy] [dilution factor] where [light] = concentration of non-isotopic metabolite, (abundance light) = total area abundance of non-isotopic metabolite, (abundance heavy) = total area abundance of isotopic metabolite, and [heavy] = known concentration isotopic metabolite.

SUPPLEMENTARY REFERENCES

1. Nemkov T, D’Alessandro A, Hansen KC. Three-minute method for amino acid analysis by UHPLC and high-resolution quadrupole orbitrap mass spectrometry. Amino Acids. 2015 Jun 10;

2. Gehrke S, Reisz JA, Nemkov T, Hansen KC, D’Alessandro A. Characterization of rapid extraction protocols for high-throughput metabolomics. Rapid Commun Mass Spectrom RCM. 2017 Sep 15;31(17):1445–52.

3. Nemkov T, Reisz JA, Gehrke S, Hansen KC, D’Alessandro A. High-Throughput Metabolomics: Isocratic and Gradient Mass Spectrometry-Based Methods. Methods Mol Biol Clifton NJ. 2019;1978:13–26.

4. Reisz JA, Zheng C, D’Alessandro A, Nemkov T. Untargeted and Semi-targeted Lipid Analysis of Biological Samples Using Mass Spectrometry-Based Metabolomics. Methods Mol Biol Clifton NJ. 2019;1978:121–35.

5. Stefanoni D, Shin HKH, Baek JH, Champagne DP, Nemkov T, Thomas T, et al. Red blood cell metabolism in Rhesus macaques and humans: comparative biology of blood storage. Haematologica. 2019 Nov 7;

6. D’Alessandro A, Fu X, Kanias T, Reisz JA, Culp-Hill R, Guo Y, et al. Donor sex, age and ethnicity impact stored red blood cell antioxidant metabolism through mechanisms in part explained by glucose 6-phosphate dehydrogenase levels and activity. Haematologica. 2020 Apr 2;

7. Reisz JA, Zheng C, D’Alessandro A, Nemkov T. Untargeted and Semi-targeted Lipid Analysis of Biological Samples Using Mass Spectrometry-Based Metabolomics. Methods Mol Biol Clifton NJ. 2019;1978:121–35.

8. Gehrke S, Rice S, Stefanoni D, Wilkerson RB, Nemkov T, Reisz JA, et al. Red Blood Cell Metabolic Responses to Torpor and Arousal in the Hibernator Arctic Ground Squirrel. J Proteome Res. 2019 05;18(4):1827–41.

9. Melamud E, Vastag L, Rabinowitz JD. Metabolomic Analysis and Visualization Engine for LC−MS Data. Anal Chem. 2010 Dec;82(23):9818–26.

10. Chong J, Soufan O, Li C, Caraus I, Li S, Bourque G, et al. MetaboAnalyst 4.0: towards more transparent and integrative metabolomics analysis. Nucleic Acids Res. 2018 Jul 2;46(W1):W486–94.

11. Nemkov T, Hansen KC, D’Alessandro A. A three-minute method for high-throughput quantitative metabolomics and quantitative tracing experiments of central carbon and nitrogen pathways. Rapid Commun Mass Spectrom RCM. 2017 Apr 30;31(8):663–73.

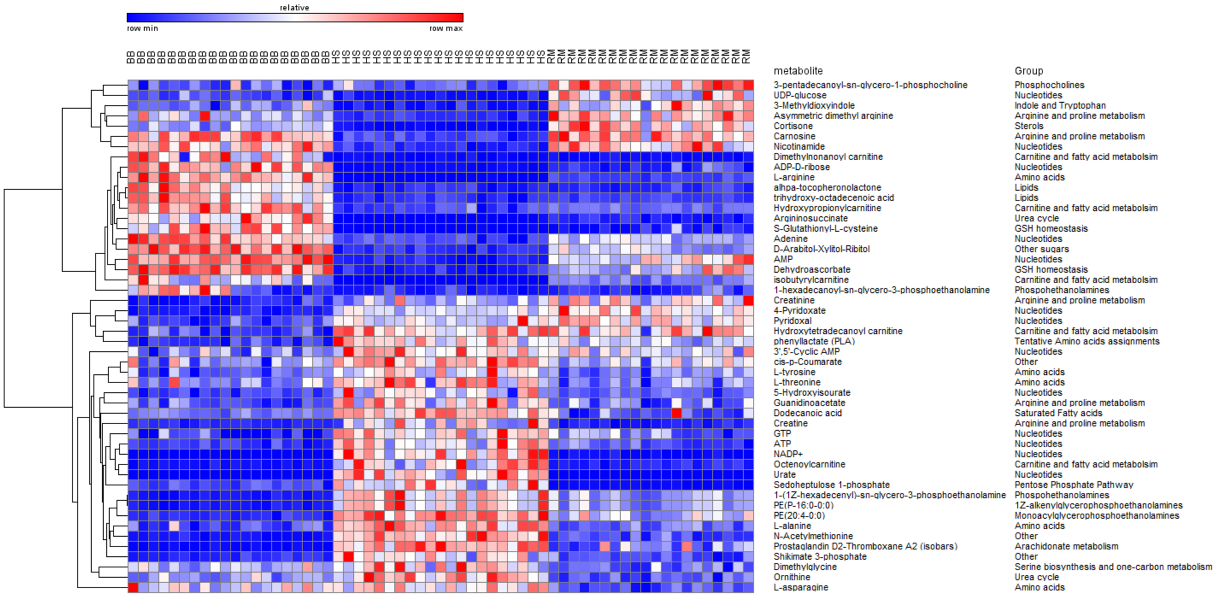

Supplementary Figure 1 – Vectorial version of the heat map in Figure 1.B

Supplementary Figure 2 – Vectorial version of the top half (RBCs) of the heat map in Figure 2.B

Supplementary Figure 3 – Vectorial version of the bottom half (sups) of the heat map in Figure 2.B

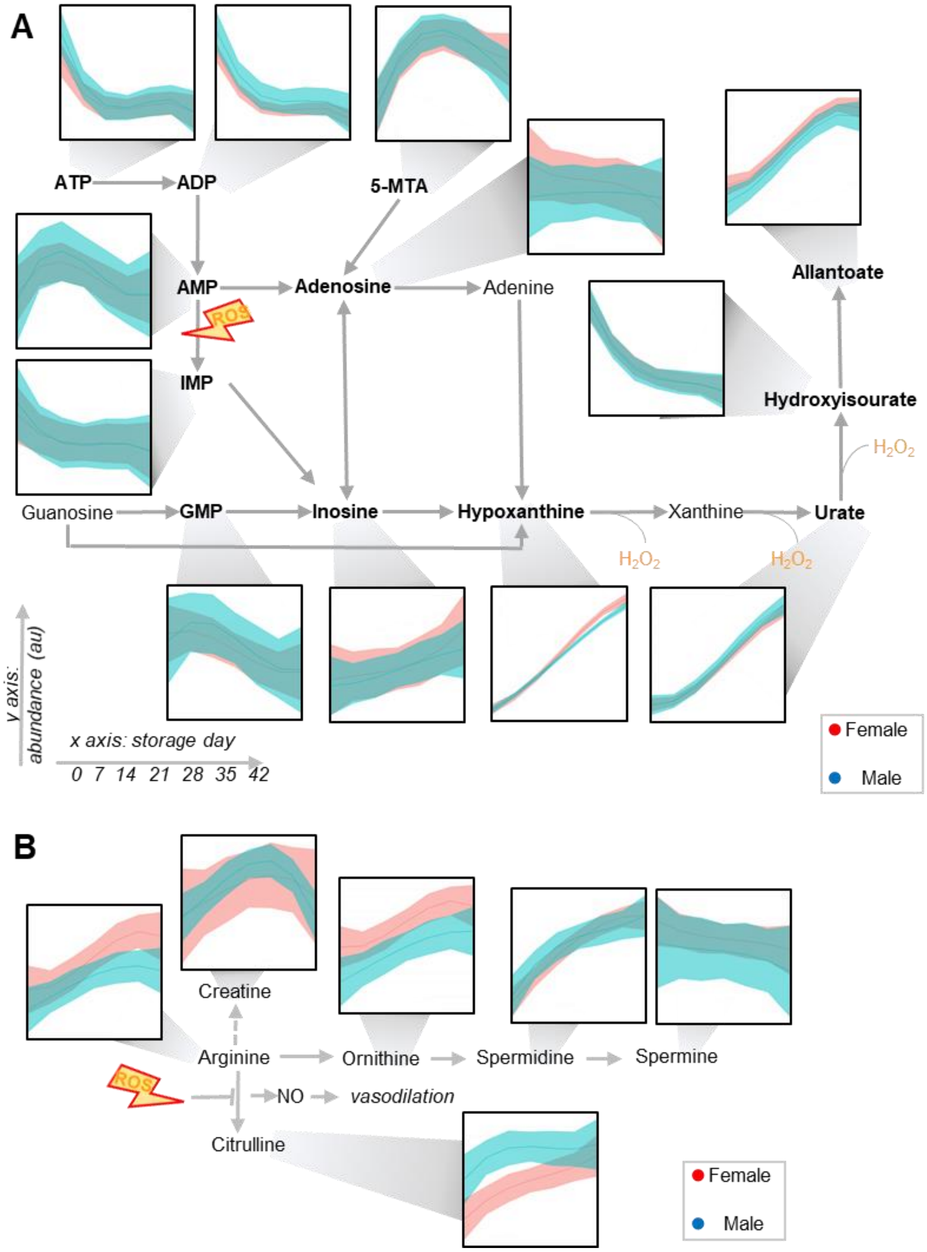

Supplementary Figure 4 – Purine (A) and arginine (B) metabolism in female (red) or male (blue) baboon (n=10 per group) RBCs from Storage Day 0 to 42. Only citrulline (**B**) showed significant differences. Line plots indicate median (n=20) ± quartile ranges for all the groups.

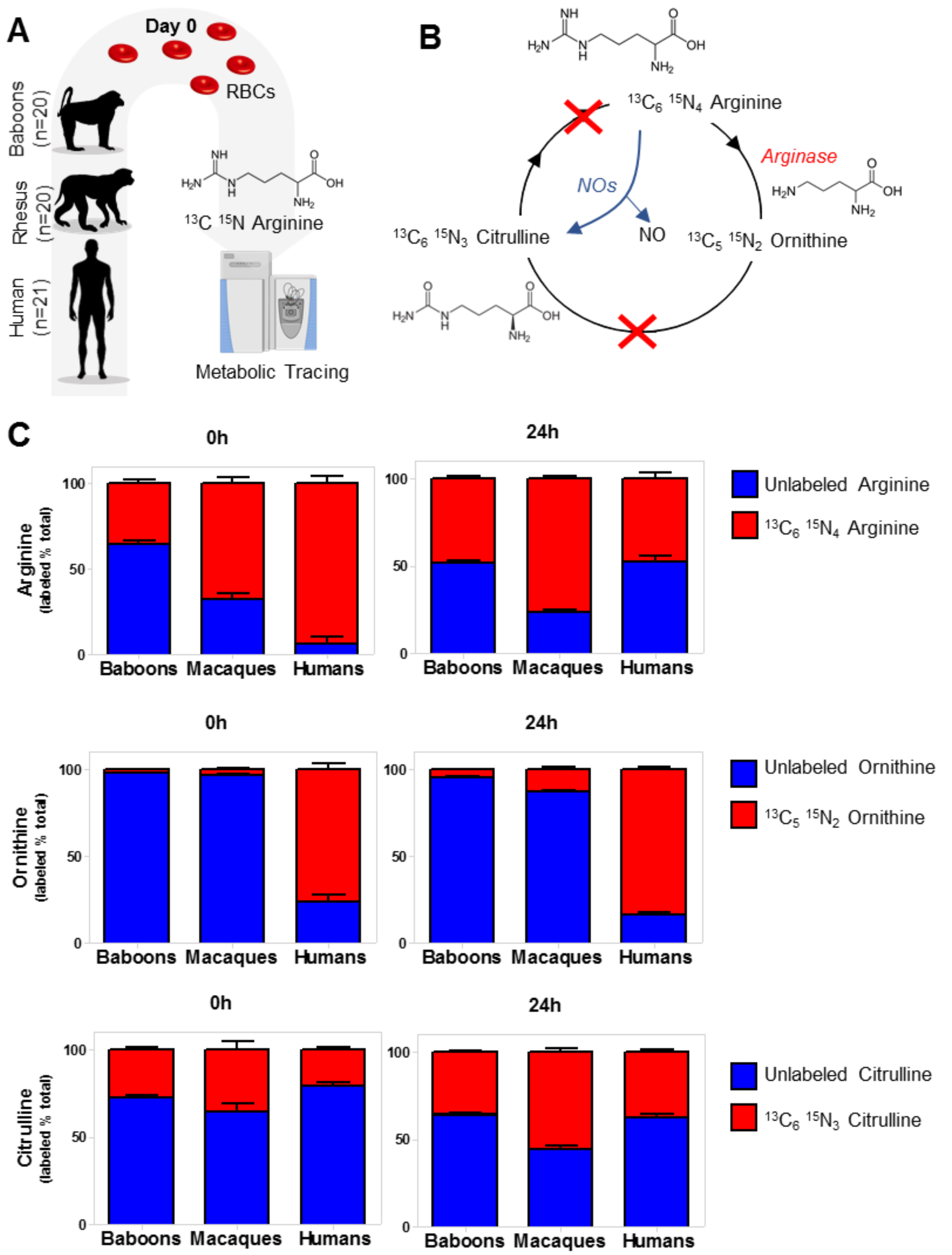

Supplementary Figure 5 – ^{13}C ^{15}N-Arginine tracing in human, baboon, and macaque RBCs. RBCs from baboons (n=20), macaques (n=20), and humans (n=21) were incubated with stable-isotope uniformly-labeled $^{13}C_6$ $^{15}N_4$-arginine for 5 min (0h) and 24hours (24h; **A**). Tracing of heavy carbon atoms into ornithine (via arginase activity) or nitric oxide synthase (but not through the urea cycle) was observed in this analysis (**B**). Specifically, we noticed that, at parity of arginine supplementation to the AS-3 (i.e., 5 mM), human RBCs had a higher percentage of labeled arginine after 5 min of incubation as compared to the NHPs (0h), although macaques had significantly higher labeled arginine after 24h (**C**). Notably, human RBCs generated a significant amount of labeled ornithine within minutes after incubation with

labeled arginine (**C**), with only minimal levels (<10%) of labeled ornithine observed in macaques at 24h of incubation. Conversely, humans showed significantly higher (>20% more) labeling in citrulline than baboons or macaques at the 24h incubation time point (**C**).

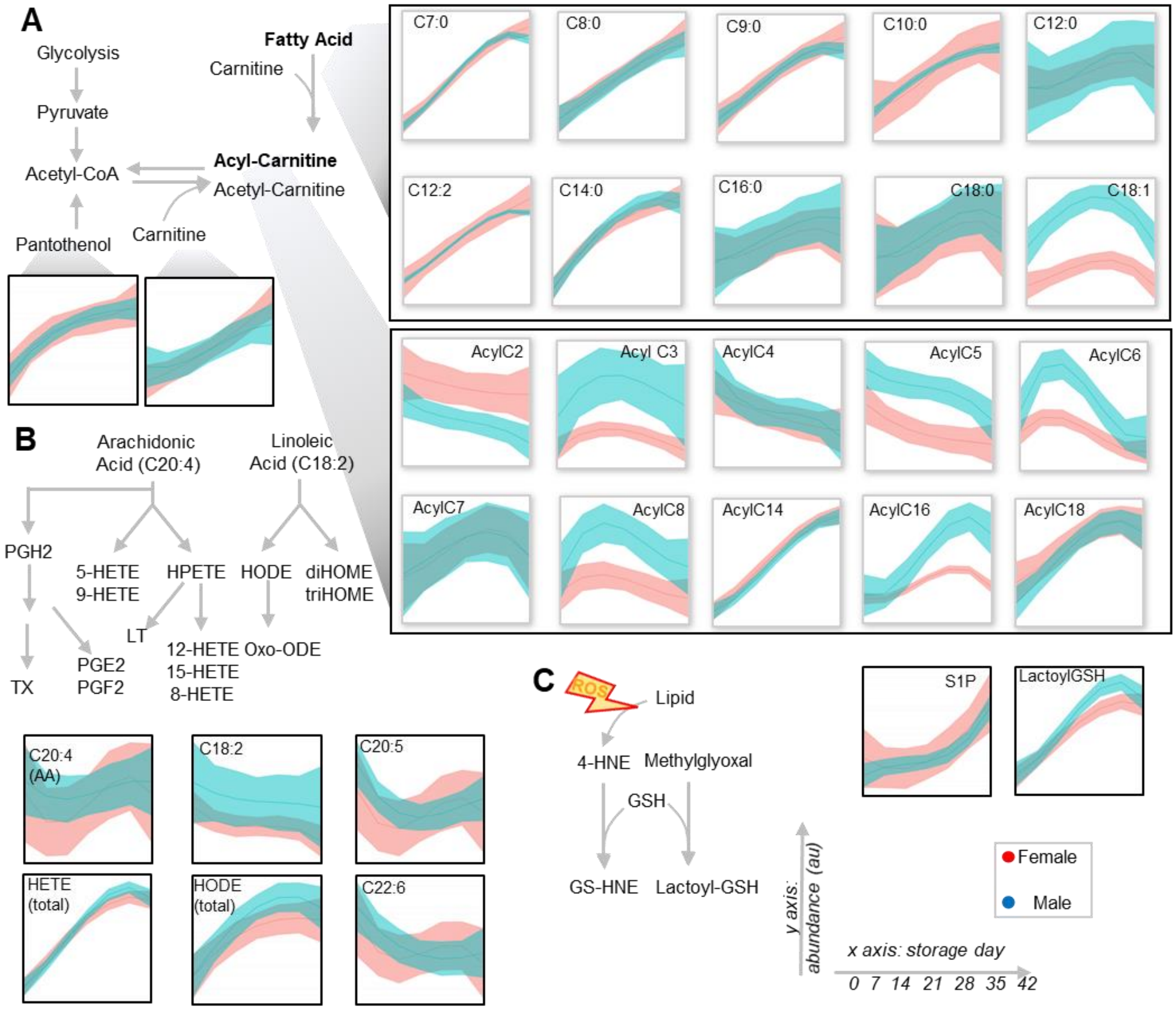

Supplementary Figure 6 – Fatty acid (top), acyl-carnitine (bottom; A), eicosanoids (B), and glyoxylate and sphingosine 1-phosphate (S1P) metabolism in female (red) or male (blue) baboon (n=10 per group) RBCs from Storage Day 0 to 42. In panels **A** and **B**, abbreviations indicate the length in carbon atoms (C) of the fatty acid chain and the number of desaturations. The prefix "Acyl-" denotes Acyl-carnitines in **A**. Line plots indicate median (n=20) + quartile ranges for all the groups.

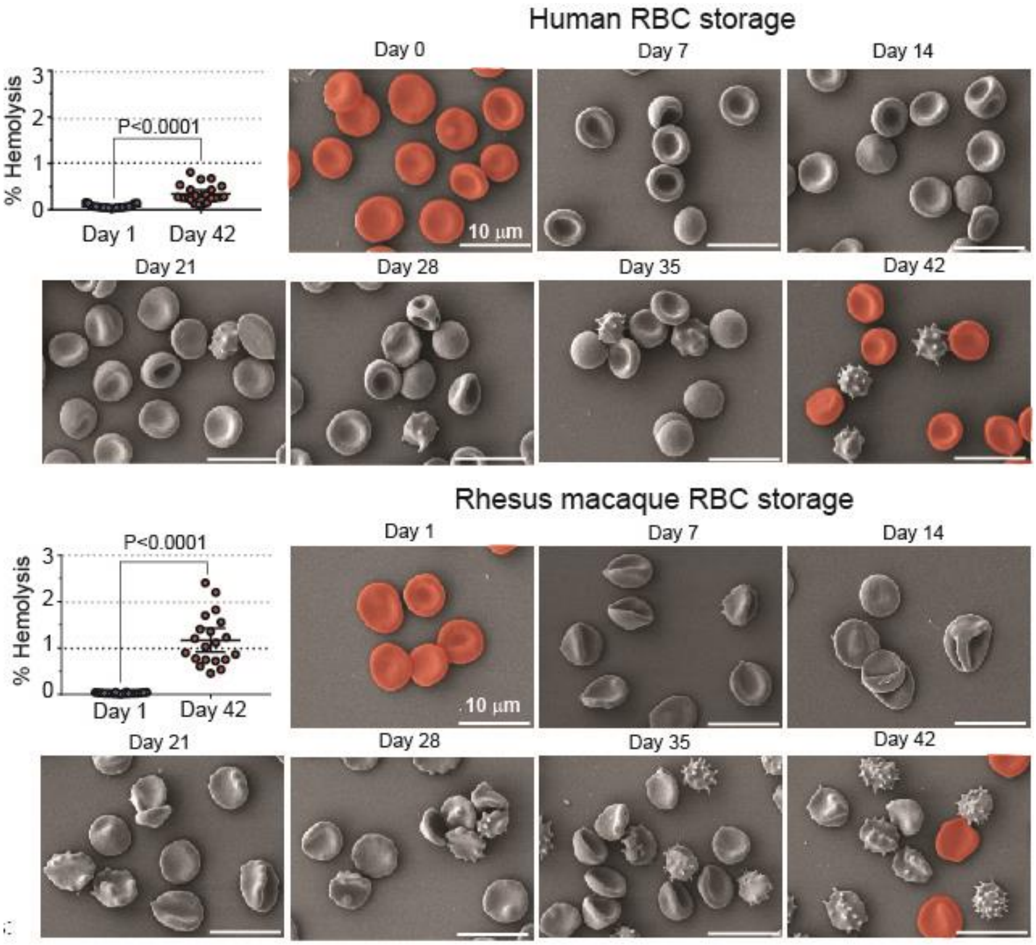

Supplementary Figure 7 – Hemolysis (Storage Day 1 and 42) and weekly scanning electron micrographs of human (top) and macaque (bottom) RBCs during standard refrigerated storage from Storage Day 1 through 42. Hemolysis data are shown as mean ± 95% confidence intervals.

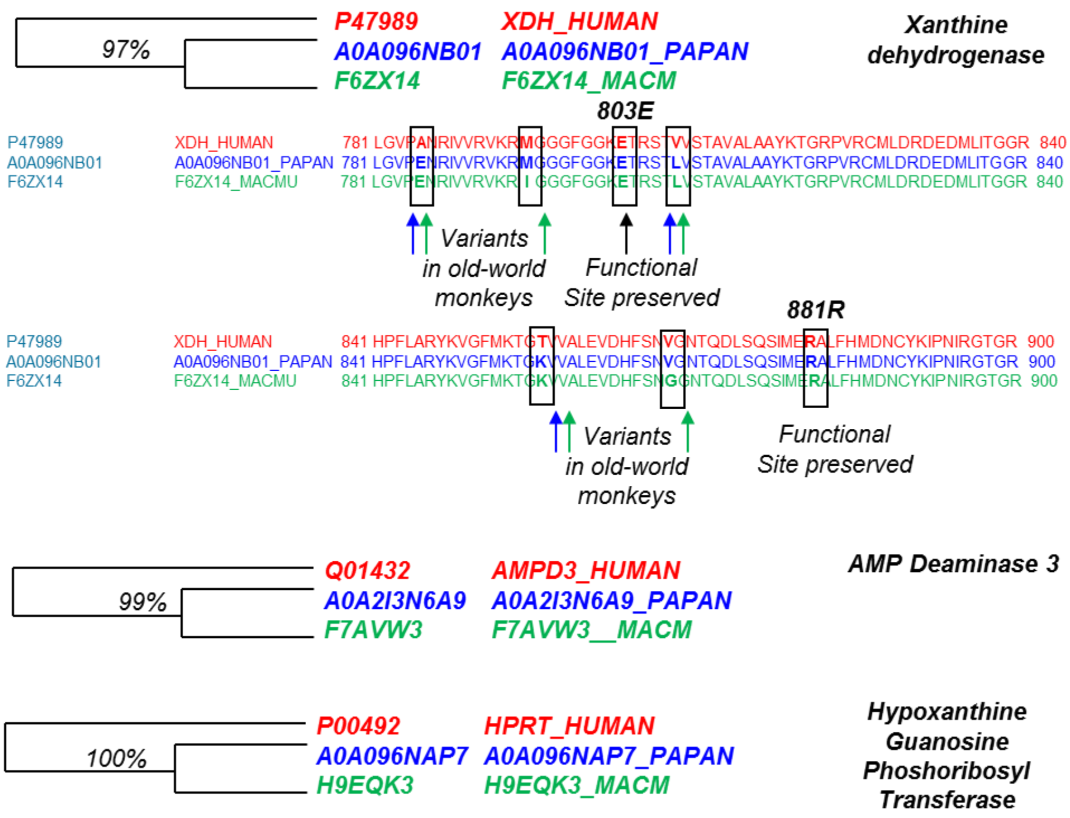

Supplementary Figure 8 – Sequence homology between human, baboon, and macaque xanthine dehydrogenase/oxidase.
