# Supplementary figures and images for: ZOOMICS: Comparative Metabolomics of Red Blood Cells From Old World Monkeys and Humans
Source: Front Physiol. 2020 Oct 23;11:593841. doi: 10.3389/fphys.2020.593841 (PMC7645159; doi:10.3389/fphys.2020.593841)

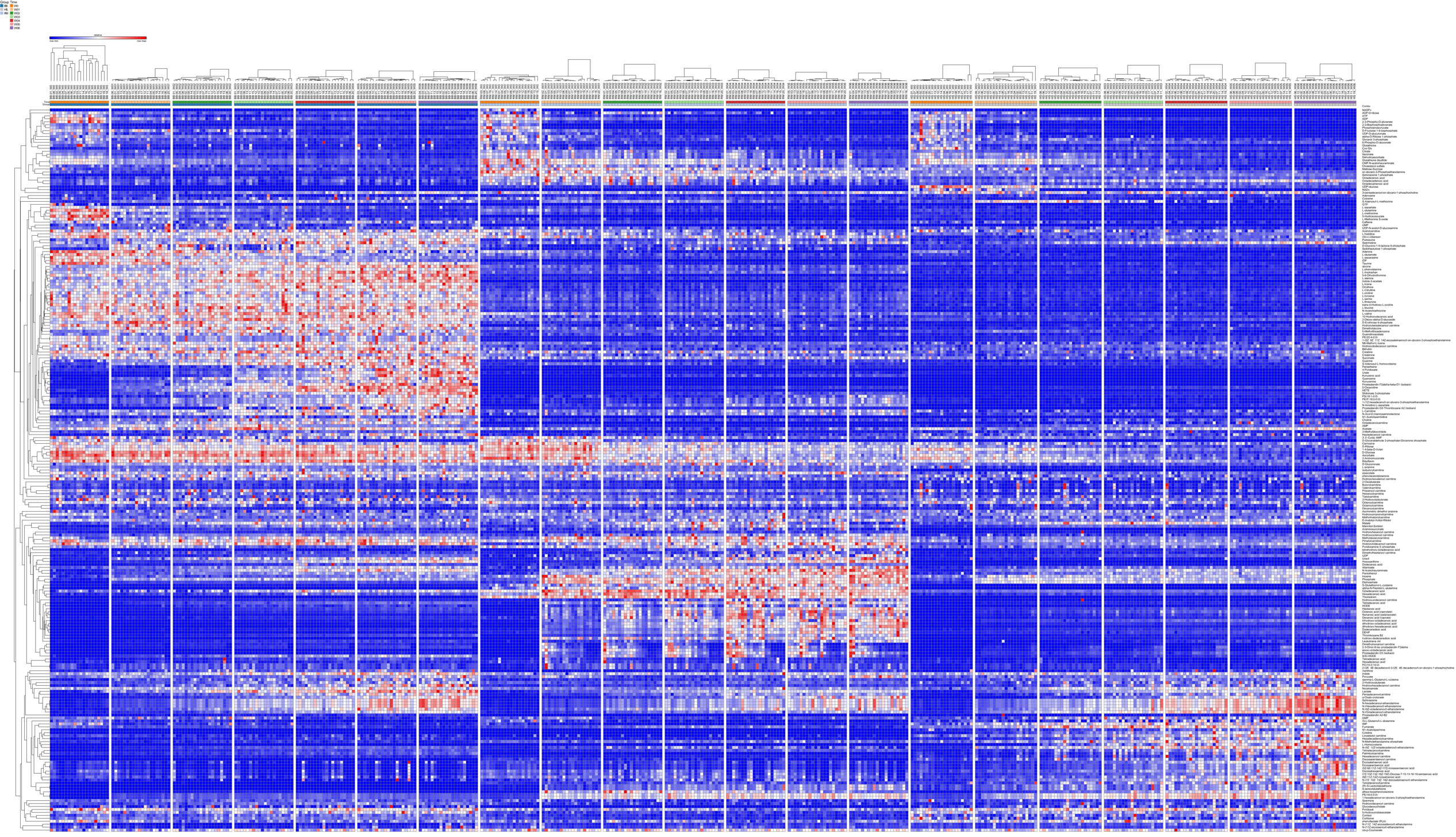

Supplement: Supplementary file 2 [file Data_Sheet_2.PDF]
