# Supplementary material for: ZOOMICS: Comparative Metabolomics of Red Blood Cells From Old World Monkeys and Humans
Source: Front Physiol. 2020 Oct 23;11:593841. doi: 10.3389/fphys.2020.593841 (PMC7645159; doi:10.3389/fphys.2020.593841)
Supplement: Supplementary file 3 [file Data_Sheet_3.PDF]

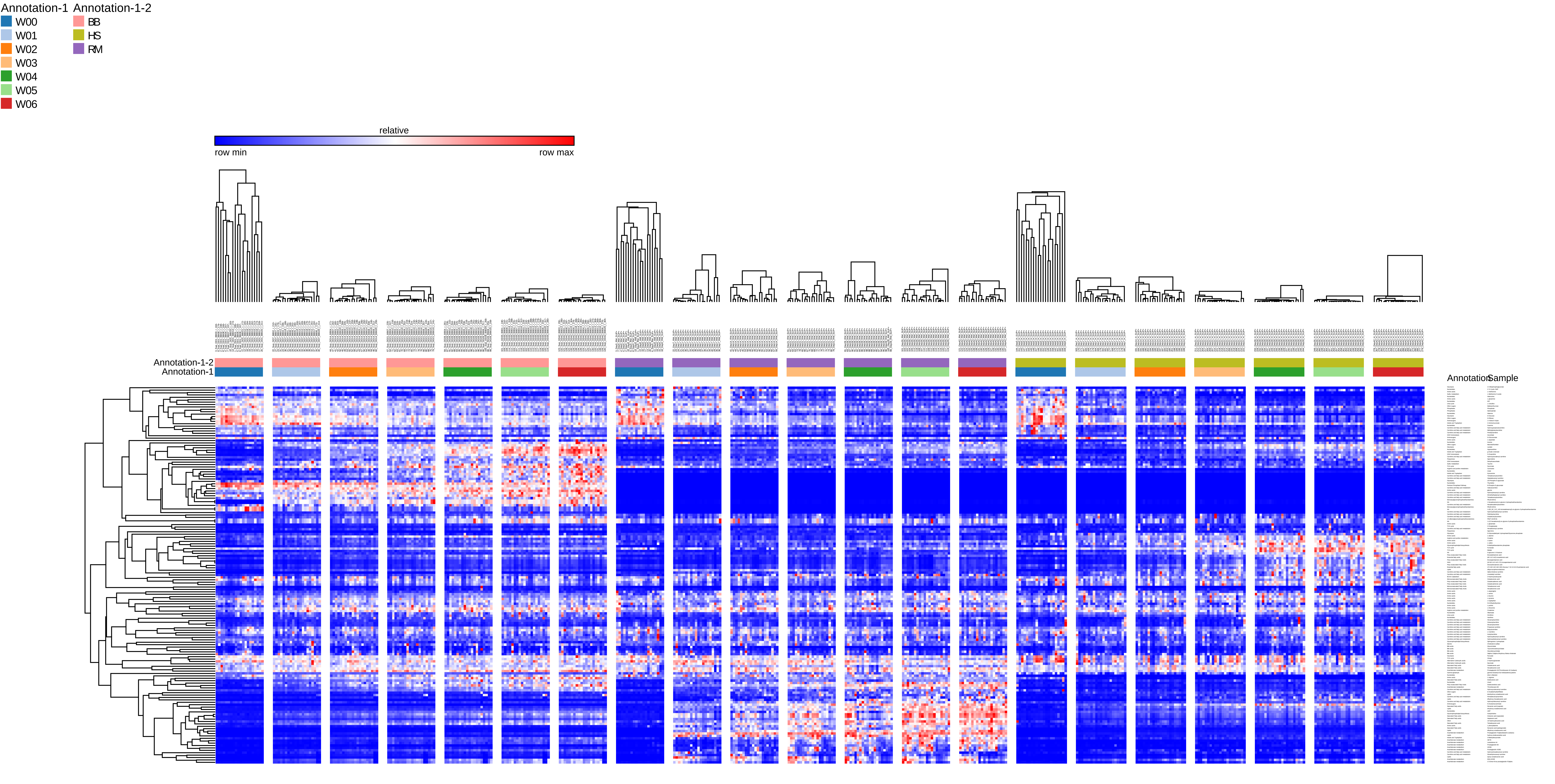

Annotation-1
W00
W01
W02
W03
W04
W05
W06
Annotation-1-2
BB
HS
RM
relative
row min
row max
Annotation-1-2
Annotation-1
Annotation
Sample
